# Mr. Hodgson's Case of Epilepsy

**Published:** 1801-04

**Authors:** W. Hodgson

**Affiliations:** Bishopwearmouth


Gentlemen,

THE following is a Caſe of Epilepſy, cured by Electricity. Should it meet your approbation, ſo as to merit its inſertion in your valuable publication, by ſo doing you will much oblige,

Your's, &c.

*Biſhopwearmouth, Feb.* 23, 1801. W. HODGSON.

Mary Hill, the fubject of the cafe, a young girl about eight years of age, had been afflicted with the complaint previous to my feeing her for three years, during which time fhe was attacked every day with three or four fits, lafting an hour or more each time; the effect fometimes being fo great as to caufe a fufpenfion of the intellectual powers for the fpace of eight weeks, accompanied with a lofs of fpeech and power of ufing her limbs. She had taken feveral medicines recommended in fuch cafes before the ufe of the machine, but entirely without benefit; then, as the laft refource, I determined to try the effect of electricity. During the firft week of ufing the remedy, the fits, which were then very fevere, were furprifingly reduced to one every other day; and from that time difappeared, only at intervals, in the fecond week, feeling a propenfity to fleep, which was fucceeded by fymptoms of an approaching fit; none, however, was the refult, and fhe has never had a return except once, about three months after, being in confequence of her father having reprehended her feverely for fome fault.— The remedy, which had been difcontinued after the ufe of it fix weeks, was again applied to with its former fuccefs; three years have fince elapfed, and fhe is now in the moft perfect health.

---

